# Modern Sanitation

**Published:** 1884-04

**Authors:** G. Frank Lydston

**Affiliations:** Late Resident Surgeon Charity Hospital, and State Emigrant Hospital and Refuge, New York City; Lecturer on Genito-Urinary Diseases, College of Physicians and Surgeons, Chicago, Ill.; 125 State St., Chicago


CHICAGO MEDICAL

JOURNAL & EXAMINER.

Vol. XLVIII.—APRIL, 1884.—No. 4.

### Original Communications.

### Article I.

Modern Sanitation. Some of Its Fallacies and Relations to the Zymotic Diseases, with Especial Reference to the Defects of Our So-Called "Modern Improvements."

By G. Frank Lydston, m.d., late Resident Surgeon Charity Hospital, and State Emigrant Hospital and Refuge, New York City; Lecturer on Genito-Urinary Diseases, College of Physicians and Surgeons, Chicago, Ill. Read before the Chicago Pathological Society, November 12, 1883.

The subject which I have the honor of presenting to the society this evening, is one in which I have always taken the deepest interest, and one, too, upon which I have had occasional opportunities to make practical observations; but when the idea of embodying the results of those observations in an article first suggested itself to me, I was somewhat at a loss for a title suitable to such a topic, and since beginning the work I have keenly realized the fact that the one selected is so broad and comprehensive in its scope, that the few ideas which I shall endeavor to present to you this evening are incapable of doing it justice. One can hardly realize the vastness of such a subject, until he has himself attempted its discussion. I must, therefore, apologize for the disappointment which may quite naturally be experienced from the comparison of the title of this paper with the subject matter which it contains.

The subject of modern sanitation is by no means a new one, but it is of such great practical importance that a presentation of new ideas in relation to it, or even a discussion of well recognized facts bearing upon it is seldom out of place, and in my estimation the time is not far distant when our medical colleges will add the teaching of at least the rudiments of practical sanitary engineering to the duties of the chair of hygiene. The knowledge thus acquired will give to the physician of the future a marked superiority over him whose learning consists mainly of ideas concerning a third blood corpuscle, or a newly discovered cell in the cortex of the brain of the dog; who, in other words, has literally been taught to "strain at gnats and to swallow camels." In order that such sanitary teaching shall be of practical benefit, and not an actual injury, considerable modification of many of the present views and customs of sanitarians will become necessary. If I were asked to name the most potent cause of sickness among the population of our large cities, and the most important influence tending to promote the dissemination of those diseases termed at the present day "germ diseases," but which, for the benefit of those who object to the army of infinitesimal organisms, we will term the "zymoses," or miasmatic affections, I should answer, "modern improvements," and I should feel that I had spoken volumes of practical truths in those two words. "But," it may be asked, "is not our expensive plumbing founded upon correct principles, and can there be anything better as a preventive of the evil effects of filth accumulations and noxious materials of various kinds?" To this I would simply answer, that the principles of modern plumbing are not only not correct, but they are radically wrong; and instead of being a safeguard against disease, they actually promote its extension.

Next to an entire disregard for sanitary matters and simple cleanliness, the plumbing of the modern mansion is the worst system that could possibly be adopted for the purpose for which it is devised. It defeats its own object, and under the present system of drainage the plumber, however skilful, is simply attempting impossibilities, and as might naturally be expected, is continually failing. I think that I am perfectly safe in asserting that there is not in Chicago to-day a perfect piece of plumbing, or a drain trap that perfectly fulfils the object for which it is intended. Our more advanced sanitarians are fast coming to realize the imperfections of our plumbing and sewerage systems, imperfections that are a necessary evil, no matter how great the outlay of time, money, and skill that may be made in the attempt to introduce perfect plumbing and drainage into our dwellings. If such a thing exists, I should like to be shown a single perfectly ventilated soil or drain pipe, or sewer, or a perfectly trapped water closet in this city. Examples may be cited in which such things are supposed to exist, and quite reasonably, too, if we consider merely their expensiveness and elaborateness of detail, but it will be found that none of them will bear searching and scientific criticism. The reason for this is quite simple, viz.: *the absolute impossibility of producing any mechanical arrangement for the purpose of preventing the escape of noxious vapors and gases into our houses, which is free from defects, and will perfectly fulfill the function for which it is devised.*

In calling attention to the fallacies of our modern sanitary arrangements, we will first consider that nowadays apparently indispensable convenience, the bath or water closet, with which our houses of even modest pretensions are generally supplied. As a rule, we find this closet upon the same floor with numerous sleeping apartments, being probably so placed in order that the unconscious occupants may have the full benefit of the expensive plumbing, with its manifold defects, and noxious emanations; in short, that they may duly appreciate the luxury of "all modern improvements," which have been, perhaps, so highly lauded as the chief attraction in that particular dwelling. Sometimes the closet is situated upon the upper floor of the building, in which situation the ventilation is possibly better, and the evil consequently somewhat mitigated, but more often it is in the middle of the house, or as in the case of our modern flats, each floor is suppliedwith a closet. But the situation is not all that is open to criticism; usually these closets are illy ventilated, and completely secluded from the sunlight. Even pure air, deprived of light and the molecular change afforded by ventilation, grows stagnant and devitalized, and its oxygen inactive*, in which condition it is decidedly unwholesome, and how much more important must be the changes in air loaded with fecal emanations and the various noxious effluvia which arise from the human body. Allowing that the closet is perfectly trapped—which in my estimation is an impossibility—there must necessarily be noxious exhalations diffused through the atmosphere while the closet is in use, and if these are not quickly removed by thorough ventilation they must necessarily undergo organic changes, which render them particularly obnoxious to the health of individuals occupying the house. This is especially apt to be the case if the closet be used by persons who are in ill health from any cause, and whose excretions are consequently abnormal. Whether the germs of disease may develop *de novo* under such unsanitary conditions is a mooted point, I, myself, however, being of those who believe in such a spontaneity. Or, lest I be understood as being a believer in spontaneous generation, I will qualify my statement by asserting that I believe that under such circumstances the innocuous organisms always present in the atmosphere in greater or less number may undergo a change, and acquire new properties by virtue of which they become infectious, and capable of transmitting disease. An apparent illustration will be cited later on.

That the specific germs of various diseases will, when introduced into a room loaded with animal exhalations, multiply rapidly and increase in virulency is, I presume, disputed by no one. Now, as for the trapping of this water closet, it has been conceded by good authorities that a perfect system of trapping has yet to be invented. No trap, however perfectly constructed, as far as

*Richardson, in his Diseases of Modern Life, calls especial attention to this point, which is quite generally ignored.

mechanical principles are concerned, can completely prevent the upward passage of the noxious volatile gases and vapors arising from the soil pipe. This may be better appreciated by considering the fact that there is always an upward pressure upon the distal side of the valves exerted by the volume of gases arising from the sewer, and in addition a continuous suction force upon the proximal surface of the trap, produced by the rarefied atmosphere of the closet. The ball valve, S. and P. water traps are alike defective, and although they may partially prevent the passage of gases when they are closed, none of them offer much hindrance to the escape of a considerable volume of gases and noxious vapors the instant the trap is pulled open. I should like to see a movable joint or valve which is so nicely adjusted and the surfaces of which are so accurately apposed that no gases can pass it. Gases which may under moderate pressure be forced into and through a granite block, are not likely to be greatly obstructed by anytrap, however nicely its joints may be made. An attempt to overcome this defect in the trap is illustrated by the S. or water trap. In this instance, the gases are supposed to be absorbed by the water, which is constantly standing in the trap; but unfortunately, the small amount of gases retained by the water is infinitesimal, as compared with the whole amount which rises from the pipes, and as a consequence we have the residue over and above the absorptive power of the water to retain such materials, simply filtered through it, and up into the atmosphere of the room, shorn perhaps of a portion of its offensive odor, but not a whit less noxious than when it left the sewer.

As I have already intimated, the air of the bath-room is always at a higher temperature than the air, gases, and vapors in the soil pipe and sewer, and there is consequently a suction force on the one hand and an upward pressure of gases against the trap upon the other, favoring a sudden influx of gases and vapor into the room, as soon as the trap is opened. No trap, it seems, can be made so perfect that this influx will not occur in greater or less amount, and there is therefore no remedy for the evil as far as the mere escape of these substances is concerned. But their noxiousness is partially remediable, for it is perfectly feasible to destroy any specific or poisonous property which such effluvia may contain. This can only be done by chemical means. The trap should be so arranged that the water will run for some time after it has been opened and closed, and its valves should be double, and disposed in such a manner that when the upper one is opened, the lower will be closed, and *vice versa*. Between the two, and in the S, if such be the form of the trap, there should be a space constantly filled with a considerable qnantity of water, and into this a solution of zinc chloride, sulphate of iron, or similar disinfectant, should be trickling continually, during the time that the stool is being used, and should run for a short time after its use has been suspended. This should convert the water which constantly stands in the trap into a solution sufficiently powerful to effectually decompose all foul gases, and to destroy all disease germs which may be present in the effluvia of the sewer. These are the indications, and it only remains for the proper mechanical principles to be applied, for their fulfillment. A recent invention called the "germicide" attempts to meet these indications after a fashion. It even goes further, and by a disinfectant (?) spray, endeavors to destroy the noxious emanations which arise during the use of the closet. The apparatus is well enough in its way, but nothing will fully meet the indications, that is not a composite part of the trap itself, for in the absence of proper mechanical principles, such a contrivance is almost worthless.

Even when constructed as I have indicated, the trap will not perfectly fulfil its function, for there must necessarily be some escape of gases during the flow of materials from the pan to the trap, and from the trap into the soil pipe, at a time when the disinfectant solution is too dilute to be of service. As for the spray mentioned, it is an attempt to "slay an elephant with a popgun," and should be replaced by free ventilation, and the admission of the sunlight, in the absence of which the processes of oxidation and chemical decomposition in nature's laboratory with difficulty occur. These suggestions have been made under the supposition that the water closet in dwellings is insisted upon, and that we are compelled to make the most of a bad bargain. We may, by following these suggestions, mitigate the evil, bu cannot entirely remove it. Would it not be better to lay th demon completely by dispensing with the closet in the dwelling, and building it as a separate establishment, not after the style of the old-fashioned privy—which is, however, in my estimation, far preferable to the modern closet, as far as its influence upon sanitation is concerned—but with the style of trap already indicated? But there is a stronger element of danger to the health of the inmates of our modern dwellings than that afforded by the water closet, *per se*, and that is the stationary wash basin.—The bath tub is usually situated in the water closet, and simply adds its evil propensities, which are identical with those of the stationary wash basin, to those of the closet proper.—Here we are absolutely helpless, as far as I can see. Practically, the man who sleeps in a room provided with one of these conveniences is in direct and most intimate relations with the sewer. His sleeping apartment is at one end of a direct channel of communication with the excreta, and refuse material of the whole neighborhood. The arrangement is much like the apparatus for the administration of nitrous oxide gas. We will let the sewer stand for the reservoir of gas, the waste pipe for the tube, and the room for the mouth piece, but instead of putting the latter over the mouth of the individual, we will most conveniently put him inside of it. The gas is already turned on, and all our victim of misplaced confidence in plumbers has to do, is to breathe as usual, and he will get his system nicely saturated with sewer gas, together with any little donations in the shape of disease germs, which may have been contributed by his sick neighbors. His sleeping apartment is the terminus of a channel of stenches and filthy vapors too numerous too mention, and of germs the most various and infectious.

Our sewers are so imperfectly ventilated, that they are constantly full of gases, which, from their close confinement and tendency to expansion, are necessarily exerting a constant pressure in all directions, and are bound to escape in the direction offering the least resistance, and consequently enter the various offshoots and soil pipes opening into the main sewer. At the end of some one of the various diverticula of these modern improvements, we have a room, the heated air of which exerts a continuous suction force, and literally draws the gases upward.* In this room we find the man to whom we alluded a moment ago, sleeping perhaps, and all unconscious of the unsavory condition of the atmosphere. The windows may be tightly closed, lest a single molecule of the lovely sewer-gas should escape. I think that nearly all of us must have noticed the gurgling produced by the waste water as it escapes from our wash basin. Now, it is not the downward suction force of the waste pipe that causes this; *it is the displacement of the gases and vapors in the pipe by the escaping fluid, and these gases, when thus displaced, rise, and escape into the room.* What better proof of my statements could be adduced? "But," it may be argued, "the room does not smell of sewer gas, and is surely not in very bad condition." Very good; the room does not smell badly, perhaps, but that is no proof that its atmosphere is in a healthful condition, or that sewer emanations are not present in greater or less amount. Now, what is "sewer gas," so-called? It is not a definite compound, but is composed of sulphuretted and carburetted hydrogen, ammonia, watery vapor, various organic matters in a state of putrefaction, and, worst of all, very often it contains the specific germs of disease.

The odorous elements of this villainous compound are perhaps the least noxious of all, and the worst cases of sickness resulting from sewer emanations, are often experienced in dwellings in which no odor is perceptible. As for the odor of sewer gas, were it not for its presence in most instances we would have no protection whatever, for it warns us of impending danger.

Why is it that there is such a continual warfare upon the varirious beneficent stenches? They are our only salvation. It would be well for some of our sanitary enthusiasts to attempt the destruction of the enemy itself, instead of wasting their time and energies in disfiguring its uniform and colors. But more of this hereafter. We will now return to our man. If you remember, we left him peacefully respiring a choice mixture of gaseous and organic substances contributed by the sewer in the middle of the street. This alone was bad enough, for, as Richardson says, "the disease *par excellence* derived from the sewer, is that con-

* *Vide* an interesting article by Prof. Max von Pettenofer on "Ground Air in its Hygienic Relations." *Pop. Science Monthly*, July, 1877.

tinued fever which is induced by the natural atmosphere of the sewer, by the inorganic products of organic decomposition, of which sulphuretted hydrogen(?) plays a leading part," but he is subjected to something infinitely worse than mere communication with the sewer. He is in direct communication with a case of diphtheria, scarlet fever, typhoid, or other similar affection, a few doors or perhaps some blocks away. The excreta from this patient are thrown into the closet, as a rule, without any attempt at disinfection, and finally reach the sewer, to add specific properties to the already great noxiousness of the composite whole which we term "sewer gas." Now, this condition of affairs has not been allowed to exist without attempts to remedy its evils, and, obviously, the most natural thing in the world would be to attempt to prevent the entrance of gas into the rooms—*i. e.*, to prevent the damming back of the gases and vapors into the dwelling, by ventilators interposed at various intervals in the course of the soil pipes. This is very often done, but almost invariably imperfectly. The ventilation is insufficient, the ventilating pipes being too small; and, above all, there is no upward current in the ventilators, and the gases, as a consequence, ignore them entirely, and pour by, and up into the house. Now, if we must have a waste and soil pipes, let them at least be ventilated as perfectly as possible. The main sewer should have open ventilators at frequent intervals, and should be frequently flushed, preferably by the method adopted in certain portions of Paris, by a continuous stream of water allowed to run in the gutters morning and night. There should be one large ventilator between the opening of the soil pipe into the sewer and the building, and there should be at least two ventilating shafts communicating with the soil pipes in the building. These should be perfectly straight, and should open at the top of the house, or preferably, into the chimney. They should either pass near the furnace or should open into a heated chamber at the top of the building, thus ensuring a draught in the pipe, and an upward movement of the noxious gases arising from the sewer, which have been intercepted as they were being dammed back into the house. *Without some such arrangement, ventilating shafts are absolutely worthless, for the gases and vapors will inevitably pass by them,*

*and instead of rising into the cold air at the top of the house, will seek the far warmer and more inviting atmosphere of the rooms at the ends of the waste pipes.*

Now, supposing we have succeeded in rendering our water closet innocuous, or nearly so, and have ventilated our soil pipes and sewers, we ought to feel quite secure, *and so we might be, if our plumbing was only perfect, but it is absolutely impossible to make it so.* The reasons for this have been set forth quite clearly by Dr. F. H. Hamilton*, and there is no disputing his conclusions. There is more security in iron pipes, according to this authority, than in lead, but they rust easily, unless protected by what is known as porcelain lining, and they are not only expensive, but very awkward in manipulation, and obviously unfitted for use where pipes of small caliber are required. In the case of leaden pipes, galvanic action is set up in the elements contained in the solder composing their joints, as a result of which they became corroded and defective in a very short time. As a consequence, no house is safe from the evils of imperfect plumbing, unless the pipes are very frequently inspected, and rarely will they remain perfect for a year. In addition, it is always difficult, and often impossible, to detect all imperfections which may exist, and prove elements of danger. Under these circumstances, there is only one means of safety, and that is, as Hamilton suggests, to remove all "modern improvements" from our residences. Let a separate building be set apart for baths, water closets, stationary wash basins, and the like, this building communicating with the main building by a well ventilated passage-way, so arranged that the door of the extension and the door of the main building can never be open simultaneously.

Quite a number of intelligent persons have already adopted modifications of this plan, or similar ones, and it is a striking fact, that some of our most pretentious private residences and hotels in the East have had their various modern conveniences, and especially the stationary wash stands removed. In Vanderbilt's new residence, I believe, they have been entirely omitted. When this plan is not followed, the next best thing to be done is to follow the plans of disinfection, free ventilation, and frequent

**New York Medical Gazette*, March 25, 1882.

and careful inspection of the plumbing, already set forth. The war cry of the sanitarian of the future will doubtless be—providing he is not compelled to practice medicine for subsistence, in which case he might quite naturally be expected to advocate the plumber's cause—"away with modern improvements!" very much as our German friends are crying: "fort mit dem spray!" but with a great deal more justice.

A very important feature of modern sanitation is the season, as well as the manner of erecting buildings. The mistake is quite commonly made of erecting buildings in winter, when the moisture in the masonry and other materials can not evaporate, and allowing them to be occupied before the walls have thoroughly dried out. It is estimated that a good brick can absorb about 10 per cent. of its own weight of water. Assuming that it only takes up 5 per cent., and allowing about 100,000 bricks to the building, and about 5 per cent. weight of water for the mortar, which is a very low estimate, we have 100,000 pounds, or 10,000 gallons of water in the composition of an ordinary brick dwelling, which must evaporate before it is fit for habitation.* These damp walls disturb the equilibrium of the bodily temperature of the occupants of the house, chiefly by rapid abstraction of heat, and as a result we have those vaso-motor disorders, and organic changes due to "taking cold," viz.: rheumatism, catarrh, bronchitis, Bright's disease, etc. I do not wish to be understood as recommending impervious building materials, for porous materials and pervious walls are absolutely necessary to keep the air of our dwellings dry and wholesome, but they must be kept dry and pure by heat and free ventilation, and no house should be occupied until it has been thoroughly dried and aired. Above all, when the house is finally occupied, the air should be kept sweet and pure, and free from sewer emanations, lest the porous building materials absorb and elaborate noxious gases and vapors. If the walls of our dwellings are saturated with water, it is obvious that all moisture over and above their capacity for absorption must either condense upon their surfaces and the bodies of those persons occupying the rooms, or remain in the atmosphere as vapor. By this vaporous condition of the atmosphere the heat of

* Pettenkofer Op. Cit.

the animal body is not only abstracted too rapidly, but the eliminative function of the skin is prevented, or at least diminished. As a result of the vaso-motor disturbances and derangement of physio-chemical changes thus induced, we have internal congestions, fever and a vicarious strain upon the mucous membranes of the respiratory and intestinal tracts and kidneys. In short, we have a cold, and with it perhaps an inflammation of that portion of the economy least capable of resisting the strain. As has been stated, these injurious effects of dampness may be prevented by free ventilation and warmth. New buildings should be thoroughly dried out by heat, and the free use of unslaked lime, before occupation.

But porous building materials have some disadvantages, the chief of which is the difficulty of disinfecting them when once they have become saturated with noxious vapors and gases, which they so readily absorb, and almost as readily give up again after they have absorbed a certain amount. Thus a building, the walls of which have once become saturated with sewage emanations, or the emanations of a large number of human beings, *i. e.*, crowd poison, will retain its poisonous properties for an indefinite time. This is especially apt to be the case with sick rooms and hospitals, which may continue to generate disease and increase the virulency of the contagion which they elaborate until they are absolutely untenable. The ordinary measures of disinfection are inefficient to correct this condition, for the reason that they are not sufficiently powerful, and do not reach the disease germs which are contained in the pores of the masonry and wood work. An instance of this kind was seen at Bellevue Hospital some years since. The building had become so saturated with poison that to be operated upon in Bellevue Hospital meant death by pyæmia. Every measure of cleansing, ventilation, and disinfection seemed ineffectual, and the only thing left would have been the entire destruction and rebuilding of the hospital if the matter had not been placed in the hands of the eminent chemist, Professor Doremus. This gentleman cleared the hospital of its inmates, and generated enormous quantities of chlorine gas in the wards, by means of the chloride of sodium, sulphuric acid, and the binoxide of manganese. The building having been tightly closed, was allowed to remain filled with the gas for some days, after which time it was again occupied, and was found to be perfectly disinfected, pyæmia being subsequently a thing of rare occurrence. The chlorine had penetrated the walls of the building and destroyed the germs of disease which had been retained and elaborated in their substance ; and it was only by the use of this powerful disinfectant, and in quantities sufficient to instantly destroy the life of any human being who might be so unfortunate as to inhale it, that these little organisms could be destroyed. Chlorine gas is especially adapted for purposes of disinfection, on account of its powerful affinity for hydrogen. Nearly all noxious vapors and gases, or those at least which emanate from the animal body, are composed largely of hydrogen, and once this is removed, they are decomposed and their noxiousness destroyed. Chlorine gas then is the disinfectant *par excellence*, and all other disinfectants are very inefficient by comparison.

Building materials vary somewhat as regards their porosity or perviousness to gases and vapors. Sandstone and brick are quite permeable. Wood absorbs noxious materials quite readily. The principal substance used in the construction of buildings, as far as its powers of absorption and permeability to gases are concerned, is the mortar or plaster. Granite and limestone are not very permeable, but such a large quantity of mortar is necessary in joining them, that this impermeability is more than compensated for. Thus it may be readily seen that any building may under favorable circumstances become unfit for habitation, and especially for hospital use, from simple absorption of poisonous materials from the air within it. In this manner, many buildings become so saturated with the contagion of different diseases, that their entire destruction by fire would be the most sensible and economical method of of disinfection. I have personal knowledge of several buildings of this kind, the relations of which to their occupants are of the utmost importance. The first of these that I will mention is the New York Maternity Hospital. This is built in the form of a low wooden pavilion, or cottage, and is about half a mile from the main building of Charity Hospital, Blackwell's Island, of which it forms a part. This building had been used for an obstetrical ward entirely, and had been in use for several years. Outbreaks of puerperal fever, at the time of my service at the Charity Hospital, were frequent, and it was my good fortune to select the waiting wards in which the women were retained until confinement was imminent, as an adjunct to my surgical service. In fact, outbreaks of fever were so frequent at the Maternity, that the waiting wards afforded a better midwifery service than the obstetrical service proper, all of the cases being delivered in tbe waiting wards during an endemic of puerperal fever at Maternity.

Now it was rather a peculiar fact that cases of fever were rare in the waiting wards of the main hospital, in which the attending physician had a general service at the same time, while they were frequent at the maternity proper, where tbere was a special corps of physicians who were not allowed to enter the general hospital wards or attend surgical operations, for fear of infection. An additional circumstance in favor of a successful obstetrical service, was that *all cases complicated by malaria, syphilis, Bright's Disease, or anything favoring puerperal complications, were at all times retained in the waiting wards.* Now, the simplest explanation of these peculiar facts is, that the maternity hospital had never been thoroughly disinfected after the development of puerperal fever, and the poison had consequently kept accumulating, until the building was absolutely unfit for an obstetrical ward. At the main hospital a large number of cases of labor only occurred during the time that there was an endemic of fever at Maternity, and consequently, at other times the opportunities for the occurrence of fever were relatively few. The maternity building has recently, I believe, been given up entirely, as it should have been long ago.* At the time of my own service, the women were in such dread of being sent to the maternity hospital, on account of the frequent occurrence of fever in its wards, that they would often conceal their pains until too late for their transference to that institution. Another even more striking instance of the pestilential properties acquired by buildings used for the shelter of large numbers of human beings, may be seen at Ward's Island,

* In a letter to the *N. Y. Med. Record* for Dec. 23, 1882, I set forth as another reason for the prevalence of puerperal fever in the maternity hospital, the indiscriminate syringing, and other meddlesome midwifery, and constant battle wlth "germs" in vogue in that institution.

New York. On this island, and under the control of the State Emigration Commission, are two large wooden structures, known respectively as the "barracks" and the "nursery." In one, indigent adult male emigrants are kept, and in the other the women and children. Cases of enteric fever arose quite commonly among the men and women, which were probably due to the general unsanitary condition of both barracks and nursery, but other diseases, excepting, of course, an occasional case of small-pox, which was immediately isolated, were rare, only because adults are not very susceptible to the contagious exanthemata. Among the children, however, the results of habitation of infected buildings were only too evident, for just as soon as a family was sent to the nursery, nearly all the children who had not had measles or scarlatina, contracted one or the other disease. Indeed, it was a common thing for one or the other gentlemen of the medical staff and myself, to speculate as to the number and variety of cases which we would receive into the children's wards from some large family sent to the "nursery."

Now it may be asked, why we did not remedy the evil, and such a question would be quite natural, coming from any one who has never had the pleasure of serving in a public institution controlled by politicians, but those who have had such service can readily appreciate the fact that protests upon the part of the staff were but a waste of breath, not to speak of the dangers of the official guillotine. Whether a hospital millennium will ever arrive, is a question, but it most assuredly never will until hospitals are placed under the absolute control of their physicians. In the instances which I have quoted, the torch would have been the only efficient remedy. For my own part, I believe that all expensive buildings, which are used for hospital purposes, should be periodically disinfected with chlorine gas. They should be so arranged that no ward can be in continuous use for more than six months, or at most a year, the pavilion or cottage hospital plan of construction being the best for this purpose, as one building can be thrown open and kept thoroughly ventilated for months if necessary, while another is being occupied. The best plan, perhaps, would be to build inexpensive structures, which can be destroyed entirely after a certain time, and rebuilt, thus doing away with inefficient disinfection, and the possibility of saturation with the poison of disease. By these means only can our houses and public institutions be prevented from becoming literal hot-beds of disease.

A very important fact relative to modern sanitation is brought forward by Pettenkofer, in his lectures upon hygiene, viz.: "The permeability of the soil to air and gases of all kinds. By a consideration of this property of the earth, one can readily understand how accumulations of filth in the cellars or basements of houses may give rise to serious results in the occupants of dwellings some distance away. Earth, which is so generally regarded as a most potent disinfectant, will absorb gases, but will not retain them necessarily, and never over a certain amount; it will filter both gases and fluids, but *it will not necessarily remove their noxious properties for evil.* This is important as showing that *local unsanitary conditions may through the medium of the porous soil and independently of the sewers produce deleterious effects in persons whose dwellings are some distance away from the site of filth accumulation.* This should be remembered, as it may serve to explain many otherwise inexplicable cases of disease. To quote Pettenkofer: "Remarkable testimony as to the permeability of the ground, and of the foundation of our houses, has been given by gas emanations into houses which have no gas laid on. I know of cases where persons were poisoned by gas which had to travel for twenty feet under the street, and then through the foundations, cellar vaults, and flooring of the ground floor rooms." To my own mind, this permeability of the soil explains the origin of some of those apparently inexplicable cases of typhoid fever in which the cesspools and closets of the dwelling are on a lower level than the well from which the drinking water is obtained; this arrangement being supposed by many to be an insuperable obstacle to the propagation of typhoid fever through the medium of the drinking water. It seems highly probable that the poison of typhoid fever may exist as a diffusive miasm—containing germs if you will—which may pass upward and in all directions from the focus of infection. If this be disputed for typhoid, it must be allowed that sewer emanations will thus diffuse, and produce an enteric fever, so closely allied to typhoid as to be almost indistinguishable from it.

In spite of many apparently absolutely inexplicable cases of typhoid fever, and the skepticism of many of our best authorities, it is still my own humble opinion that every case of the disease originates in some local unsanitary condition, which may operate through the medium of either the air respired or the water drunk by the affected individual. The air of a foul cellar may give rise to typhoid fever in the people occupying the house above, through their respiratory tracts, quite as readily as can pollution of their drinking water by filth. That the poison of typhoid consists in living organisms or germs, has, it seems to me, been demonstrated;* but I believe that the innocuous organic particles or germs, which are everywhere present in the atmosphere, may, under certain circumstances, acquire new properties, by virtue of which they are capable of transmitting diseases of a specific character, the type of disease being modified by the peculiar local conditions present. The typhoid, or, for that matter, the typhus poison, may arise spontaneously, not from "spontaneous generation," which we hold to be impossible, but by the spontaneous development of specific properties of infectiousness in organisms previously harmless. The best remedy for the danger of the entrance of noxious vapors and gases into houses through the medium of the ground, is to build the walls of the foundations of our dwellings double, leaving a wide area between them, which will also answer the purpose of protection against damp, as is suggested by Fothergill.† This area should, of course, be perfectly ventilated. In addition, the cellar floor should be double, a space being left between its layers, which communicated with the area, and is thereby freely ventilated. As an additional precaution against damp, the outer walls and the floor of our cellars might be constructed of glazed tile or concrete, as advised by the authority just quoted. This arrangement, with free ventilation between the walls of our buildings, would probably be as near

* Vide theory formulated by Desplat and supported by Klebs and Eberth. *Bulletin Gen. de Thérapeutique*, June 30, 1883.

† "Maintenance of Health."

an approach to sanitary perfection, as far as the evils which it is designed to combat is concerned, as could well be devised.

There is a peculiar feature of modern sanitation that sometimes leads me to wish that disinfectants had never been invented. I refer to the tendency on the part of the municipal authorities—and, for that matter, many physicians as well—of some of our large cities, to fight existing or impending epidemics, or even sporadic cases of disease, very much after the fashion of a Chinese method of warfare—with "stinkpots." Now, this plan of action would doubtless be very effective, if our little disease germs were only vulnerable to stenches; but, alas! they are not, for if they were, extinction of the whole race of germs, bacteria, micrococci, etc., would be an easy matter at the hands of some of our friends. But for this tendency to attempt the suffocation of germs. Certain people, both among the laity and in our own ranks, entertain the delusive idea that all that is necessary to destroy the noxious properties of filth and decomposing matters, whether animal or vegetable, is to give them the odor of carbolic acid, or some other vile smelling compound. In so doing, what do they accomplish? They simply substitute one vile smell by another which is often more intolerable than the original which it is intended to destroy, and which is particularly objectionable in that it lulls them into fancied security. I saw this exemplified in New York City in 1878, during the epidemic of yellow fever in the South. Great and well-founded apprehension was felt by the people lest the disease should appear in the city, and indeed every element necessary to its development was present, all that was necessary being the application of the torch to the tinder, as the city was abominably filthy, and the season a very hot one. Now, obviously the proper course would have been to clean the city thoroughly, and remove, or, preferably, destroy all the rubbish and filth. But, instead of doing this, the authorities cleaned(?) up the filth in a slip-shod way, which merely stirred it up into renewed activity, and then spilled carbolic acid around in a promiscuous manner until the atmosphere was almost intolerable. I verily believe that high-colored urine must have been epidemic at that time, so redolent was the air with carbolic acid, the quantity being only a little short of sufficient to kill the people, and a great deal short of the amount necessary to destroy germs. Thanks to this thorough disinfection (?), perhaps, but more likely through the interposition of Providence, the city escaped the yellow fever; but in 1881 * a fine harvest was reaped in the shape of an epidemic of typhus fever, which was clearly traceable to local unsanitary conditions, in spite of the efforts to shift the responsibility upon immigration and importation of the infection †. The first cases of the disease observed entered the Charity Hospital, Blackwell's Island, and were severally under the care of Dr. Merriam and myself, and in a very few weeks after their detection we were actually overrun with typhus, there being at one time about 160 cases of the disease upon the island. Upon investigation, the origin of the disease was found to be what was known as the Shiloh lodging-house, in the slums of the city. This establishment was formerly a church, but had fallen into decay, and had been converted into a sort of tramps' retreat, a charge of 5 cents being made for a bed upon a bench, sleeping under the benches being said to be free, providing the lodger lived till morning and chopped a stipulated quantity of wood. The lodging-house did a thriving business, and the advantages for the spontaneous development of disease were very fine. The majority of the cases of typhus originated here, fully 150 cases being traceable to either direct or indirect contagion from it. Cases soon sprung up, however, in various other localities, the Italian tenement houses being especially prolific. As far as this epidemic was concerned, I had occasion to feel cordially thankful toward the city fathers and their stinkpots, for the opportunities for clinical and pathological observation thus presented were exceptionally fine, and in fact I have little curiosity to gratify in regard to typhus at the present time, and I might add in regard to cholera, also, although I have had no experience with it. I think, however, that some of those present who have had experience with these diseases will echo this sentiment. We will hope that our own exceedingly conscientious and indefatigable(?)

* In 1880 I believe, the city was in such a filthy condition, that mass meetings of physicians were held to urge precautionary measures on the part of the authorities, This mitigated the evil somewhat, so that the above mentioned epidemic of typhus fever was much milder than it would otherwise have been.

† Liverpool was, I believe, the alleged source of infection.

municipal authorities will not adopt the odoriferous and fallacious plan of prevention that I have described.

But what is to be done in lieu of such a course? Let the streets, alleys and sewers of our city be thoroughly cleaned, and the accumulations of filth removed and destroyed as far as possible, by burning or quick lime, or in certain instances let it be removed a long distance, and spread in thin layers upon the soil, and allowed to dessicate. Let the sewers, soil and drain pipes be frequently flushed, and thoroughly ventilated, and all cesspools and collections of stagnant water be opened up and cleaned, after which allow them to remain exposed to the air and sunlight. After all has been done that pure water, good ventilation and the sun's rays can accomplish, it will be ample time for "disinfectants," which are well enough in their way, if they are not used to disguise the effects of filth emanations, when the cause should be removed. The application of disinfectant drugs is ordinarily too much like the administration of many so-called "specifics" in cases of disease. *Let the cause be removed, and there will be little use for such substances.*

As I have stated, the use of the stinkpot is not entirely confined to either Chinese warfare, or modern municipal or lay sanitation, but it is exceedingly popular with many intelligent physicians. How often we may enter a sick room and find the windows tightly closed and the light carefully excluded, and be greeted with the odor of chlorine, carbolic acid, or similar substances. There is no characteristic sick room smell, and in the language of the physician and friends, the apartment is perfectly "disinfected." Disinfected by what? On looking carefully around we find on the window sill, under the bed, or in the various utensils about the sick room, little dabs of chloride of lime, or squirts of carbolic acid, which might be termed homœpathic attempts to kill an elephant. And this is disinfection! Save the mark! Would it be possible to convince this physician and these people in attendance that it would require chlorine gas or carbolic acid enough to kill the whole household to even benumb one of the little germs which act as carriers of disease? Or would it be possible to demonstrate to them the fact that there is an increase in the tenacity of vitality as we descend in the scale of organic life? If so, a great and philanthropic work of charity would be accomplished. Let the physician try to destroy one of these little germs. He will find that they are destroyed, or as some claim, merely benumbed, by a 1 to 2 per cent. solution of carbolic acid. Let him now, if possible impregnate the air of the sick room with carbolic acid to a strength of 2 per cent., and what would become of his patient? It is safe to say that he would soon be pretty thoroughly prepared for a pathological collection. Or let him, as an experiment, try the destruction of the germs in the living blood, in any given case of disease, *a la* Declat, and note the effect of three or four ounces of carbolic acid in the circulation. Now remove the disinfectants (?) from this close sick room, and what do we observe? An indication previously unobserved on account of the stench of the disinfectant, viz.: a fetid and characteristic odor, perhaps of a special character, peculiar to the disease from which the patient is suffering, but in any event disagreeable and unhealthful, and which says in unmistakable language: "Open the windows and let in the sunshine and fresh air." This is a progressive age, and it is to be hoped that he timely prevention of epidemics, and the evil effects of infectious miasms, will be one day managed upon common sense principles, and combated by nature's disinfectants which oxidize and destroy, instead of by disinfectant drugs, whose chief claims to consideration lie in the intensity of the stench which they are capable of producing. Let us have less carbolic acid and more hydric oxide, sunshine and "God's oxygen."

125 State St., Chicago, Oct. 1, 1883.

